# An Experimental Inquiry Concerning Some Points in the Vital Processes of Assimilation and Nutrition

**Published:** 1851-09

**Authors:** N. S. Davis

**Affiliations:** Prof. of Principles and Practice of Medicine, and Clinical Medicine, in Rush Medical College; Member of the American Med. Association; one of the Physicians to the Illinois Gen. Hospital, &c., &c. Chicago, Illinois


ARTICLE II.

*An Experimental Inquiry concerning some points in the Vital Processes of Assimilation and Nutrition. Read before the American Medical Association at its Annual Meeting in Charleston, S. C., May,* 1851. By N. S. DAVIS, M. D., Prof. of Principles and Practice of Medicine, and Clinical Medicine, in Rush Medical College; Member of the American Med. Association; one of the Physicians to the Illinois Gen. Hospital, &c., &c. Chicago, Illinois, 1851.

The following experiments and observations constitute part of a series undertaken for the purpose of ascertaining, first: The influence of different kinds of food and drink on the functions of Calorification and Respiration, as indicated by the changes of temperature and the variations in the quantity of carbonic acid gas thrown off during expiration, in the healthy condition of the human system; and second, the changes which the several constituents of the blood undergo in their relative proportions during the passage of that fluid through the secreting organs and some of the non-secreting structures of the human body.

According to the prevailing Physiological doctrines concerning Digestion, Respiration, and Calorification, all alimentary substances are divided into two great classes, viz: the *nitrogenized* and non-nitrogenized or carbonaceous. Those belonging to the first class, are supposed to be used principally for nourishing the tissues; while those of the second, are consumed in supporting the functions of respiration and calorification. Admitting the correctness of these positions, we should reasonably infer that an individual fed exclusively on carbonaceous aliment would maintain a higher temperature and emit a greater proportion of carbonic acid gas with the expired air than when confined to a diet altogether nitrogenous. To test the correctness of this view by direct experiments constituted the object of the first series of observations which will be detailed in a subsequent part of this paper.

Closely connected with the prevailing doctrines concerning the nature and uses of different alimentary substances, is the composition of the blood, and the origin of its several proximate constituents, and their respective uses in the animal economy.

The same chemico-physiological views which have led to a division of aliments into nitrogenized and non-nitrogenized, have also regarded the latter class as the source of carbonic and lactic acids, water, &c., in the blood, the formation of which generated the caloric necessary for maintaining the temperature of the body; while the former or nitrogenized class, furnished the albumen, fibrin, and corpuscles or cell-structures, constituting the plastic materials necessary for supplying the waste of the tissues. And most Physiologists agree with Dr. Carpenter, in considering albumen as the primary form to which this class of aliment is reduced by the digestive processes, and that the conversion of albumen into fibrin is a further assimilative change—a step nearer to a state of organization. Thus, says Carpenter, "albumen is always the starting point; since the filbrinous elements of organized tissues are reduced, by the solvent powers of the gastric fluid, to the same form with the unorganized coagulum of the albumen of the egg. The conversion of albumen into fibrine, therefore, is the *first* great step in the process of nutrition, by which the materials supplied by the food are made to form part of the living tissues of the body; and it is the one to which the term *Assimilation* may be most appropriately applied." Many facts, however, are to be met with not easily reconciled to the view so ably maintained by Dr. Carpenter and others. And it was with the hope of throwing some additional light on the subject, that I instituted the analyses which constitute the second series of experiments detailed below.

*First Series.*—In this series of observations my first object was to ascertain the *temperature* of the body in a state of health, at different periods of the day, and under the influence of an ordinary mixed diet of animal and vegetable food; and coincidently therewith, the relative proportion of carbonic acid exhaled with the expired air. For this purpose I chose a healthy man, aged 34 years, whose habits of life were regular and strictly temperate, and noted his temperature as indicated by a reliable thermometer, the bulb of which was placed under the tongue, and allowed to remain five minutes with the mouth closed around it. Immediately after noting the temperature, eight cubic inches of expired air was collected in a graduated tube or small jar, inverted over mercury. From the mercury bath, the jar was removed to one of fresh lime water, and allowed to remain until absorption ceased. The height to which the lime-water rose in the jar indicated the quantity of carbonic acid which had been removed from the given quantity of expired air. These observations were repeated six times each day, viz: at 7½ A. M., half an hour before taking food; between 10 and 11 A. M., from two to three hours after breakfast; at 12½ P. M., immediately before dinner; at 3½ P. M., two and a half hours after dinner; at 5½ P. M., immediately before tea; and 8 P. M., two and a half hours after tea.

The temperature of the room in which all the observations were made did not vary at any time more than two degrees from 50° of F., and the mercury and lime-water baths were always in the same room. The following is the average result of 16 days' observations under an ordinary mixed diet, viz:

| | 7½ o'clock, A. M. | 10½ A. M. | 12½ P. M. | 3½ P. M. | 5½ P. M. | 8 P. M. |
|---|---|---|---|---|---|---|
| Av. Temperature, | 94° F. | 96° F. | 95°2 F. | 96°7 F. | 95°2 F. | 96° F. |
| Highest, | 94°5 F. | 97°3 F. | 96° F. | 97°3 F. | 95°5 F. | 96° F. |
| Lowest, | 93°7 F. | 95°6 F. | 94° F. | 96°3 F. | 95° F. | 96° F. |
| Average of Carb. Acid in 8 Cubic Inches of Expired Air. | 12-16 of a Cubic inch, or 1 part in 10.6. | 11-16 C. I. or 1 to 11.6. | 11½-16 C. I., or 1 to 11.1. | 14-16 C. I., or 1 to 9.1. | 14-16 C. I., or 1 to 9.1. | 13-16 C I, or 1 to 9.8. |

Two interesting inferences are fairly deducible from this table, viz: First, that the temperature of the body is uniformly from one or two degrees higher during the active stage of digestion, that is, about two hours after eating, than after the digestive process is fully completed. Second, that the temperature of the body and the relative proportion of carbonic acid gas in expired air, do not bear a uniform ratio towards each other. The greatest amount of carbonic acid being exhaled between the hours of 3 o'clock P. M. and 6 o'clock P. M., and then diminishing until 10 o'clock A. M., of the following day; while the temperature uniformly rose from one to two degrees during every regular period of digestion, reaching its greatest height about two hours after dinner. My next object was to continue the same observations under the influence of a diet as strictly *carbonacious* as was consistent with healthy digestion. For this purpose the same individual was confined for three days, to a diet consisting exclusively of rice starch, boiled, and white sugar, eaten freely three times a day at the usual hours. The observations during these three days were continued with the same apparatus and under the same circumstances as before, and the results are seen in the following table, viz:

| | 7½ o'clock, A. M. | 10½ A. M. | 12½ P. M. | 3½ P. M. | 5½ P. M. | 8 P. M. |
|---|---|---|---|---|---|---|
| Av. Temperature, | 93°4 F. | 95°7 F. | 95° F. | 96° F. | 94°7 F. | 95°2 F. |
| Highest, | 93°7 F. | 96° F. | 95°1 F. | 96° F. | 95°5 F. | 95°2 F. |
| Lowest, | 93° F. | 95°5 F. | 95° F. | 96° F. | 94° F. | 95°2 F. |
| Average of Carb. Acid in 8 Cubic Inches of Expired Air. | 11½-16 of C. Inch, or 1 part in 11.1. | 12½-16 C. I., or 1 in 10.2. | 14-16 C. I, or 1 in 9.1. | 13-16 C. I., or 1 in 9.8. | 12-16 C. I., or 1 in 10.6. | 12-16 C I., or 1 in 10.6. |

Two things will strike the reader while comparing this with the preceding table. First, the average temperature of the system throughout the day ranges about half a degree *lower* than under a liberal mixed diet of vegetable and animal food. Second, the average amount of carbonic acid contained in the expired air is very nearly the same for the entire day, but instead of appearing in the smallest proportion between the hours of nine and twelve A. M., and the greatest proportion between three and six P. M., as in the first table, we find these extremes between six and nine A. M., and twelve M. and three P. M. Another variation which does not appear in the table, but which was fully verified by repeated trials, consisted in the fact that the temperature as indicated by the thermometer under the tongue, more quickly attained its maximum height after eating and began again to decline, than under the influence of a mixed diet.

After a few days interval, during which an ordinary mixed diet was allowed, the same individual was confined for three days to a diet exclusively *nitrogenous*, consisting of *albumen* of *eggs*, from which the yolk was wholly excluded, eaten in liberal quantities three times a day, at the hours previously mentioned, and once a day a very small quantity of dried beef, from which care was taken to remove as far as possible every vestige of fat and cellular tissue. The following is the result, viz:

| | 7½ o'clock, A. M. | 10½ A. M. | 12½ P. M. | 3½ P. M. | 5½ P. M. | 8 P. M. |
|---|---|---|---|---|---|---|
| Av. Temperature, | 95° F. | 95°6 F. | 95°8 F. | 96°2 F. | 95°3 F. | 95°6 F. |
| Highest, | 95° F. | 96° F. | 95°9 F. | 96°5 F. | 95°5 F. | 95°8 F. |
| Lowest, | 95° F. | 95°3 F. | 95°7 F. | 96° F. | 95° F. | 95°5 F. |
| Average of Carb. Acid in 8 Cubic Inches of Expired Air. | 14-16 of a Cubic Inch, or 1 part in 9.1. | 12-16 of C. I., or 1 in 10.6. | 12½-16 C. I., or 1 in 10.2. | 12-16 C. I., or 1 in 10.6. | 12-16 C. I., or 1 in 10.6. | 13-16 C I., or 1 in 9.8. |

In glancing over this table, we are struck at once with the uniformity of temperature maintained throughout the entire day, there being a variation of only a little more than *one* degree from the lowest point before breakfast to the highest, two hours after dinner. While in both the previous tables the difference between the same hours is but little less than *three* degrees. And yet, the mean temperature for the entire day is nearly the same in all.

It being in the first under mixed diet, - - 95°5
In the second under an exclusive carbonaceous diet, 95°
In the third, under an exclusive Nitrogenous diet, 95°6

The proportion of carbonic acid exhaled from the lungs, as seen in the third table, also differs in two respects from the two preceding. First, the proportion is more uniform throughout the day, varying only from 1 part in 9.1 to 1 in 10.6 of the expired air. Second, the period when the carbonic acid was greatest is almost the reverse of that in the first table, being greatest late in the evening and early in the morning, instead of early in the afternoon. This will be seen more readily by glancing at the following table:

| | | | | |
|---|---|---|---|---|
| Average proportion of Carbonic Acid in Expired Air under the influence of a mixed diet during whole day, . . . | 1 | cubic inch | in | 10.24 |
| Highest average between 3 o'clock P. M. and 6 P. M., . . | 1 | " | " | 9.1 |
| Lowest average between 9 o'clock A. M. and 12 M., . . . | 1 | " | " | 10.6 |
| Average for whole day under Carbonacious diet, . . . . | 1 | " | " | 10.17 |
| Highest average between 12 o'clock M. and 3 P. M., . . . | 1 | " | " | 9.1 |
| Lowest average between 6 o'clock A. M. and 9 A. M., . . | 1 | " | " | 10.9 |
| Average for whole day under Nitrogenous diet, . . . . | 1 | " | " | 10.11 |
| Highest average between 6 o'clock A. M. and 9 A. M., . . | 1 | " | " | 9.1 |
| Lowest average between 3 o'clock P. M. and 6 P. M., . . | 1 | " | " | 10.6 |

A comparison of these averages with the average temperature of each day under the different systems of diet, will lead to the same conclusion as the inspection of each individual table. That is, the absence of any uniform correspondence between the actual temperature of the body and the quantity of carbonic acid exhaled from the lungs. On the contrary, it would seem that the temperature is uniformly greatest during the middle or active period of digestion, and diminishes in proportion to the length of time which elapses before a fresh supply of food is taken. Hence the lowest temperature was always in the morning before breakfast, when about 14 hours had intervened since taking food the previous evening.—While the proportion of carbonic acid exhaled reached its maximum under the carbonaceous diet about 12 M., under the mixed diet about 3 o'clock P. M., and under the nitrogenous, not till 7 o'clock the following morning.

These variations, both in regard to temperature and expired carbonic acid, are doubtless owing chiefly to the difference in the digestibility of the several classes of food used. Thus it was very evident, from the feelings of the individual subjected to experiment, that the well boiled rice starch was easily and quickly digested and removed from the stomach, accompanied by a correspondingly rapid rise and subsequent fall of temperature, with the maximum proportion of carbonic acid at mid-day. The digestion of the mixed diet was more protracted, the rise of temperature a little slower and longer sustained, and the maximum proportion of carbonic acid three hours later in the afternoon. The diet of albumen was very slow of digestion, being sensibly felt in the stomach from the end of one meal to well nigh the commencement of another, and consequently the rise of temperature was so gradual and protracted as to present comparatively little variation during the entire day, and the maximum proportion of carbonic acid was not exhibited until late in the evening or early next morning.

But the most interesting question connected with these observations, is that which relates to their bearing on the commonly received doctrines in relation to the uses of the two classes of diet, called Carbonacious and Nitrogenous. The Chemico-Physiological doctrines in regard to diet, nutrition, respiration, and animal heat, first extensively and systematically promulgated by Liebig, were so simple and plausible as to meet an almost unparalleled popular reception, and with various modifications to become speedily incorporated, as received truths, into almost all the Physiological works of the day. These doctrines, as has been previously stated, assign to the carbonaceous articles of food, including oils or fats, starch and sugar, the office of furnishing carbon and water to the blood and fat to the tissues, from which result carbonic acid in expired air and heat to the whole system; while the nitrogenous articles, including vegetable and animal albumen, fibrin, &c., were more especially or exclusively used to supply the corresponding elements of the blood, and nourish the tissues of the body. Notwithstanding these doctrines have met with so general a reception, both within and without the ranks of the profession of medicine, they rest, so far as I can discover, entirely on their plausibility, aided by considerations of the most general and ill-defined character. Such as the allegation, that man eats more fat and carbonaceous substances in winter than summer, in cold countries than warm; that all the constituents of the blood and tissues are found in the food, and consequently digestion is a process of solution and not of transformation, at least, so far as regards the albumen, fibrin, &c.; and that, as starch is convertible into sugar, and sugar, with the addition of oxygen, into carbonic acid and water out of the body, they therefore undergo the same changes when taken as food into the body, &c., &c. But I am not aware that up to the present time, any one has attempted to demonstrate their truth by direct experiments, except so far as the observations embodied in the foregoing tables may be considered such an attempt. And these, so far from demonstrating their truth, militate very strongly in the opposite direction. Indeed, should they be corroborated by further and more extended observations of the same character, we shall be forced to renounce much of the doctrines briefly alluded to, notwithstanding their captivating simplicity. If the chief uses of the carbonaceous articles of food, consist in furnishing fat and water to the blood, carbon to the respiratory process, and caloric to the whole system, then an abundant, if not exclusive supply of these articles, should both elevate the temperature of the body and increase the proportion of carbonic acid in the expired air; while a deprivation of them, should cause a rapid diminution of both. No propositions could be more clear than these. And yet, according to the experiments already detailed, performed with all the precautions to ensure accuracy which I could devise, no such results were obtained. It may be thought that three days were not long enough to afford a fair test; but if we remember that the lowest estimates place the amount of carbon exhaled at *eight ounces* every twenty-four hours, making 24 ounces of solid carbon during the continuance of each series of our observations, and then observe the contrast between an abundant or even excessive supply in the form of carbonaceous food, and an entire deprivation of this class of aliment, we certainly should have good reason to expect a marked variation in the result. For if it might be supposed the carbon already in the blood, would be sufficient to keep the proportion of carbonic acid in the expired air up to the ordinary standard for a time after the supply of the corresponding class of aliment had been cut off, this surely could not be continued, without a well marked diminution, until more than twenty ounces of solid carbon had been exhaled. But the third table given above, not only shows no diminution in the average proportion of carbonic acid exhaled while under a strictly nitrogenous diet, but the details of each days' observations included in the table, show full as large a proportion of carbon in the expired air during the last day as the first; and this, too, when less than a single ounce of any food other than albumen and water had been taken during the whole three days. Such a result taken in connection with the full maintainance of the temperature of the system, renders it at least doubtful whether the nitrogenous and non-nitrogenous articles of diet have any such separate and distinct uses in the animal economy as have been assigned to them. We should by no means consider the foregoing experiments sufficient to enable us to draw therefrom established and reliable conclusions, but should they be corroborated by further extension and repetition, they will show most conclusively that animal heat corresponds with the activity of absorption and nutrition, while the expired carbonic acid is a product of the decomposition of tissues rather than of any particular class of food. And hence that both are kept up to their full healthy standard, as well by nitrogenous as carbonaceous food, so long as the digestive apparatus is capable of dissolving either or both with facility. These views are still further corroborated by our experiments with Alcohol, which, from its highly carbonaceous character, is regarded by Liebig, and others, as one of the most efficient supporters of combustion, and consequently of animal heat, that we possess. Prout had long since demonstrated that Alcohol taken into the system, instead of furnishing carbon for the respiratory process, speedily and decidedly diminished the exhalation of that material from the lungs ; and his observations have since been abundantly confirmed by M. M. Bouchardat and Sandras. But neither of these gentlemen took any measures to test the actual variations of temperature in the system while under the influence of the alcohol, and the accompanying diminution in the quantity of exhaled carbon. And hence Liebig attempts to obviate the force of any objection founded on the results of the experiments of Prout and Bouchardat, by alledging that "the increased formation of *water*, which will occur when Alcohol is the combustive material, compensates for the diminution in the amount of carbonic acid expired, and thus the normal amount of heat may be generated." For the purpose of supplying this defect, the following experiment was performed on the same individual, and with the same apparatus as in all the previous cases.

At 9 o'clock, P. M., three and a half hours after supper, the temperature as indicated by the thermometer under the tongue was 95°, and the proportion of carbonic acid in the expired air 1 to 13. The pulse was 80 per minute, which was about the normal standard for the individual. Four ounces of strong brandy were now taken. At the end of 45 minutes, the pulse was increased to 90 per minute, the temperature unaltered, remaining precisely at 95°, but the proportion of carbonic acid in the expired air was diminished to 1 part in 14.2. At the end of one hour, the pulse had fallen to 85 per minute, the temperature still remained at 95°, but the proportion of carbonic acid in the expired air had still further diminished to 1 part in 16. At the end of two full hours, the pulse had returned to 80 per minute, the mercury failed to reach 95°, being not more than 94°8, while the proportion of carbonic acid in the expired air had perceptibly increased. At 45 minutes past 11 o'clock, 2¾ hours after taking the brandy, the temperature had fallen to 94.5, and the proportion of carbonic acid in the expired air had increased to 1 part in 12.5. This experiment was repeated a second time with precisely similar results. It will be seen that it fully corroborates the observations of previous investigators in regard to the diminution of the quantity of expired carbonic acid, while it at the same time proves that there is not only *no increase* of temperature, but a subsequent decided diminution. If it be asked, how these results are to be reconciled with the well known good effects of Alcohol in sustaining the vital energies in certain low or typhoid forms of disease, we confess our inability to answer, on the supposition that it is a simple *combustive*, or heat-generating agent. But does the defect, supplied by Alcohol, in the forms of disease alluded to, consist in simple defective or insufficient calorification?

A careful review of all those cases or forms of disease in which Alcoholic stimulants prove beneficial, will conclusively show that the fundamental defect is in the function of *innervation*, and that whatever failure there may be in the actual temperature of the system, it is secondary or consequent on the arrest, partial or complete, of the molecular or organic actions, which if not dependent on, are at least more or less influenced by the nerves of organic and animal life. And hence the beneficial effects of Alcohol in all such cases, can be much better explained, by supposing it to act directly on the nervous tissue, for which it is well known to possess so strong an affinity. By thus increasing the innervation, the organic actions are sustained, nutrition recommences and animal heat is generated—and that, too, without any *combustion* of the Alcohol, or anything approaching to the nature of that process. Such an explanation is also perfectly consistent with the effects of the Alcohol on a healthy system, as shown by the experiments alluded to above.

Without presuming to draw any conclusions from the incomplete investigations detailed above, it may not be improper to state as the result of all our researches thus far, that the doctrine of *combustion* in the animal economy, by which is meant the appropriation of certain alimentary substances to the nourishment of the tissues, and certain others to the *direct* support of respiration and animal heat, by being resolved into carbonic acid and water in the blood, rests on nothing but a purely theoretical foundation. On the other hand, the phenomena of both health and disease, unite with the experimental inquiries hitherto instituted bearing on this subject, in pointing us to the conclusion that the *carbonic acid* of the respiratory process, like the secretions from the skin and kidneys, is a true product of the decomposition or metamorphosis of the structures of the body, while the *temperature* depends directly on those changes which take place in the nutritive and organic actions.

This view compels us to suppose that all digestible substances, whether carbonaceous or nitrogenous, are assimilated and appropriated with more or less facility to the nourishment of the solid textures of the body. But it by no means precludes the idea, that certain articles of food may generate an excess of either carbonaceous or nitrogenous elements in the blood, and that such elements may serve to increase the amount of excretion from either the lungs or kidneys, without having previously constituted any part of the solid tissues, and without undergoing any process really analagous to combustion. But it was not so much my intention to draw Physiological conclusions as to state such facts as I had thus far been able to demonstrate. Hence I shall hasten to the second department of this paper.

*Second Series.*—The blood, as the common fluid from which all the animal tissues receive their nourishment on the one hand, and all the secreting organs and surfaces receive the materials for their several secretions on the other, has long been a prominent subject of study both by the physiologist and the physician. And yet the prevailing views in regard to the origin and uses of its several proximate elements are, as has been already stated, far from being satisfactory or well settled. Notwithstanding the most patient research, and the many analysis of blood which have been made, no one has yet satisfactorily distinguished those ingredients which are truly *nutritive*, from those which are incapable of affording nourishment, or have served their purpose, and are hence on their way to some one of the excretory outlets of the system. It is generally considered, for instance, that the waste nitrogenous matter resulting from the constant change going on in the organized tissues of the system, finds its chief exit through the kidneys in the form of Urea, Uric Acid, &c., but no one has yet satisfactorily pointed out the *form* it assumes while passing from the tissues to the kidneys. It surely cannot be in the form of Urea, for if so, the quantity is so great that its presence in the arterial blood would always be easily detected. And Simon's suggestion that it is the extractive matters of the blood from which the nitrogenized constituents of the urine are derived, is simply an opinion without proof. There are very few opinions in Physiology more universally assented to, especially by British and American physiologists, than that the albumen and fibrin of the blood are its chief *nutrient* elements; the latter being especially regarded as presenting the highest degree of assimilation, bearing, according to the expression of Carpenter, the same relation to organized tissue, that the "*spun yarn* does to the *woven fabric*." And yet if this is so, why do we find fibrin in its full proportion, or even in excess in protracted Anemia, in the wasting hectic of Phthisis, and in all acute inflammations, after the patient has perhaps been many days without nourishment of any kind? The ordinary explanation, which consists in attributing the increase of fibrine in these cases, to the more rapid oxydation (or assimilation, if you please,) of the albumen, in consequence of the greater rapidity of the circulation and respiration, is very unsatisfactory; first, because no such increased rapidity of either circulation or respiration, takes place in many of the cases to which the explanation has been applied; and second, because it is very difficult to conceive how more oxygen can gain access to the blood, when half of the pulmonary tissue is, perhaps, rendered impermeable by tubercular deposits and vomica, or when the access of air to the lungs is greatly diminished by an extensive pulmonary or laryngeal inflammation. Neither is the view taken by Simon,* who attributes the elaboration of the fibrin to the dissolution or metamorphosis of the red corpuscles of the blood, and who,

*It is necessary to remind the reader that this essay was prepared before the recent views of Simon, as stated in his Lectures delivered at St. Thomas's Hospital, London, during the summer session of 1850, had been seen by me. In those lectures Simon has arrived at conclusions much more nearly in accordance with those presented in the latter part of this paper; though without instituting any new experiments or analysis in support of them. It is gratifying, however, to find myself both anticipated and sustained by an authority so justly eminent.

consequently, asserts that the increase of fibrin is in proportion to the rapidity of the consumption of such corpuscles, any less liable to objections. And the rule which he lays down as the necessary consequence of this explanation, viz: "that the *amount of fibrin must always stand in an inverse ratio* to that of the blood-corpuscles," is by no means sustained either by his own analytical tables, or those of Andral, Gavaret, Rodier, and others. Indeed, the tables he has given on pages 205 and 206 of his work on the Chemistry of Man, illustrating the influence of blood-letting or the composition of blood, not only destroy the theory adopted by Simon, but furnish an additional objection in the way of considering fibrin as the chief nutrient element of that fluid. These tables, compiled from the analysis of Andral, Gavaret, Rodier, and Simon, show very conclusively that blood-letting increases the relative proportion of water and fibrin in the blood, while its corpuscles and all the other solid constituents are diminished. Now if we suppose fibrin to be a highly elaborated product, resulting from the metamorphosis of the red-corpuscles, it is certainly difficult to conceive how the direct abstraction of such corpuscles, by bleeding, should add to the relative quantity of fibrine left in the blood. On the contrary, if the one results from the natural maturation and dissolution of the other, we should suppose that the direct abstraction of the latter, without such dissolution, would necessarily be accompanied by a diminution of the former. The rapid increase of the relative proportion of water after bleeding, is by universal consent, attributed to the rapid absorption of water from the various tissues and surfaces of the body; and if we suppose fibrin to be present in the various tissues as a waste product, the maintainance of its full proportion would be satisfactorily accounted for in the same manner as the water.

Again, it would be reasonable to infer, that the blood, in a perfectly healthy state of the system, when the digestive powers were active and the supply of food abundant, would present a full or predominant relative proportion of those ingredients which result directly from the function of assimilation and are designed to nourish the textures; and it is then that we always find the blood-corpuscles, the albumen, the fatty matter, &c., most abundant.

On the other hand, when such diseases occur as are accompanied by an entire disrelish and consequent discontinuance of food, together with an active capillary circulation and rapid emaciation or waste of textures, we should expect to find those elements of the blood which are derived from a waste of the organized structures decidedly increased in their relative proportion; more especially so if those diseases were also accompanied by a diminution of the more important excretions, as is the case in almost all the acute inflammations. And what are the facts as revealed on almost every page of Pathological investigation? Simply, that these are the very diseases and circumstances under which the blood exhibits its largest proportion of fibrin and salts—especially the former. While on the other hand, all those diseases characterized by diminished innervation and consequent sluggish capillary circulation, and in which absorption and emaciation are much less rapid—such as Typhus, Scurvy, &c.—the fibrin remains normal in its proportion or diminishes, while the corpuscles and albumen remain but little or not at all diminished.

The foregoing considerations, with many others that might be mentioned, led me long since to doubt very much the correctness of the generally received opinions in regard to the origin and uses of the fibrin, as well as some of the other constituents of healthy blood. Whether the fibrin be truly nutritive in its character, or whether it be a product of the first step in the process of metamorphosis or waste of tissues, we should expect in health to find it nearly equal in the general mass of venous and arterial blood—in the first case perhaps slightly in excess in arterial, and in the latter, in venous blood. If, however, it be a product of the first step towards decay or metamorphosis of tissues, and therefore destined for excretion, we should expect the blood, in those veins which return the circulating fluid from the organs engaged in its excretion, to contain decidedly less of this element than either that of the arteries leading to such organs, or of the veins leading from non-secreting or muscular parts of the body. On looking over the analysis of Andral, Gavaret, Lecanu, Shultz, Denis, Becquerel, Bodier, Lehman, Simon, and others, I find but one attempt to institute an analytical comparison between the quantity of fibrin in the veins returning blood from secreting organs, and that found in the arteries. And I nowhere find the relative composition of the blood in the veins of an actively secreting organ, compared with that in the veins returning from non-secreting or muscular parts. There have been numerous analysis of veinous blood in comparison with arterial, but without any reference to the particular parts from which the veinous blood was returned. The one exception to which allusion was just made, was by Simon, who procured the blood from the Renal veins, the Hepatic veins, and the Aorta of a horse, and subjected it to careful analysis, particularly in reference to the quantity of *fibrin* contained in each specimen. The results are given on page 139 of his work on the Chemistry of Man, and are as follows, viz:

| Renal Vein. | | Hepatic Vein. | | Aorta. | |
|---|---|---|---|---|---|
| Water, | 778,000 | Water, | 725,000 | Water, | 790,000 |
| Albumen, | 99,230 | Albumen, | 130,000 | Albumen, | 90,300 |
| Fibrine, | ,000 | Fibrine, | 2,500 | Fibrine, | 8,200 |
| Whole solid matter, | 222,000 | Whole solid matter, | 275,000 | Whole solid matter, | 210,000 |

This presents truly a striking result; the blood returning from two of the largest secreting organs in the body is found to contain far less *fibrin* and *water*, and more *albumen* than that from the Aorta. But there are two circumstances which render this analysis unsatisfactory. First, the horse from which the blood was taken was not healthy, and in a *starved* condition. Second, the quantity of blood obtained from the Renal veins (being only 50 grs.) was insufficient to determine accurately the proportion of fibrin.

To obviate these objections, and at the same time add another important element to the comparison, I procured a large, healthy, and active dog, and procured some blood from the Renal vein, the Illiac vein, and the Illiac Artery, in the following manner: The dog was stunned by a blow on his head, the abdomen being quickly laid open, a ligature was passed around the Renal vein near its entrance into the ascending cava, and on puncturing the vein, 590 grains of blood flowed readily into a clean cupping glass. A ligature was next passed around the Illiac vein, and on puncturing it 771 grains of blood were easily collected in another cup. The Illiac Artery, being now of easy access, was punctured, and the blood flowed in a full pulsating stream, and was received into a third cup, to the amount of 1816 grains. Up to this time the dog continued to breathe slowly, and the circulation went on vigorously; but in attempting to procure some blood from the Hepatic vein, the diaphragm was cut through, the breathing ceased, and the hepatic vein was found so empty that a satisfactory amount of blood could not be obtained. The left ventricle of the heart was then laid open and a fourth specimen of blood, to the amount of 425 grains, was obtained from its cavity. All these specimens of blood were most carefully analyzed, following in all respects the method recommended and so long practiced by Andral, and Gavarret, with the single exception of the mode of separating the fibrin. For accomplishing this most accurately, I fully agree with Mr. Bence Jones, preferring to allow the blood to coagulate perfectly, and then enclose the whole clot in a clean, firm linen cloth, and wash it with distilled water until the red-coipuscles are entirely removed. From several trials I am satisfied that this method separates the fibrin more perfectly than the whipping or stirring with sticks, as practiced by Andral. There being nothing peculiar in the other steps of the analysis, it would be useless to detail them here. The results in tabular form are as follows, viz:

| | Blood from Left Ventricle. | Bld. from Iliac Artery. | Bld. from Iliac Vein. | Bld. from Renal Vein. |
|---|---|---|---|---|
| Whole amount analyzed, | 425 grs. | 1816 grs. | 771 grs. | 590 grs. |
| Solid matter after separating fibrin, | 78,2 | 341,0 | 145,0 | 115,0 |
| Water, | 345,9 | 1471,0 | 624,0 | 474,0 |
| Fibrin, | ,9 | 4,0 | 2,0 | 1,0 |
| Albumen, | 46,5 | 180,9 | 69,0 | 58,1 |
| Red-Corpuscles, | 34,0 | 150,1 | 71,0 | 54,4 |
| Salts, | 2,0 | 10,0 | 3,0 | 2,5 |

To make the differences in the above analysis more easily appreciated, I have reduced the several items to their relative proportion in 1000 parts of blood, as follows, viz:

| | Blood in the Left Ventricle in 1000 parts. | Blood from the Illiac Artery in 1000 parts. | Blood from the Illiac Vein in 1000 parts. | Bloom from the Renal Vein in 100 parts. |
|---|---|---|---|---|
| Water, | 812,0 | 812,2 | 811,9 | 803,4 |
| Red-Corpuscles, | 81,8 | 82,5 | 92,7 | 92,2 |
| Fibrin, | 2,1 | 2,2 | 2,5 | 1,7 |
| Albumen (fat and extractive matter not separated,) | 99,4 | 98,1 | 89,5 | 98,5 |
| Salts, | 4,7 | 5,0 | 3,9 | 4,2 |
| | 1000,0 | 1000,0 | 1000,5 | 1000,0 |

The very slight variations exhibited in the columns representing the two specimens of Arterial blood, are doubtless owing wholly to the slight inaccuracies incident to all analytical processes. But the difference between the two specimens of Venous blood, or between either of them and the Arterial, are striking and highly important. Thus the blood from the Illiac vein, returning mostly from a muscular and non-secreting part of the system, exhibits a higher proportion of *fibrin* and red-corpuscles, with a much lower proportion of albumen, than the Arterial blood; while that from the Renal vein, returning from an organ secreting copiously a highly nitrogenous liquid, exhibits a marked diminution in the proportion of *fibrin* and water, with an increase of red-corpuscles, the albumen remaining the same as in the blood from the arteries. These results so well correspond with those obtained by Simon, as already quoted, that but little doubt can be entertained of their correctness. And they point, with a force little short of actual demonstration, to the conclusion *that the firrin of the blood is the direct result of the waste or metamorphosis of the organized tissues*, finding its exit from the system in the nitrogenous constituents of Urine, Bile, &c., instead of being the most perfectly assimilated agent destined for nourishing such tissues. Indeed, how else is it possible to explain the fact that the blood returning from an active excreting organ, like the kidney, contains less fibrin than arterial blood, while that returning from non-secreting or chiefly muscular structures, where we should suppose the greatest amount of fibrin would be used up if it were nutritious, contains a proportion exceeding that sent through the arteries to the same structures? The conclusion thus arrived at from analysis of the blood returning from different organs and tissues, also affords a far more satisfactory explanation of the increase of fibrin in diseased conditions of the system, than has been afforded by any of the more commonly received doctrines in regard to that constituent of the circulating fluids. Thus if we grant that the fibrin is derived from the decay or waste of tissues, instead of constituting assimilated matter for their nourishment, its accumulation in the blood in all inflammatory diseases, when the supply of food is cut off, the tissues wasting rapidly, and, at the same time, the nitrogenous excretions diminished, is easily understood. So, too, its existence in a full or even excessive proportion, in Aenemia, Phthisis, or any other morbid state characterized by an active capillary circulation and a predominance of the decomposing or wasting process over the nutritive, is explained with equal facility. This conclusion also explains why the Urea appears as a constant and uniform ingredient in the Urinay Secretion, under the influence of all classes of diet, and even while life continues, after all diet is discontinued, and yet can never be detected during health in quantities above a mere trace in the blood itself. If the fibrin is derived from the waste of tissues, and is excreted in the form of Urea and other nitrogenous constituents of the excretions, we should, a priori, expect such excretions to continue, not while this or that diet was continued, but so long as the organized structures continued to undergo metamorphosis, and the secreting organs themselves continued in a condition to act. And such, observation long since demonstrated to be the fact. I do not wish to convey the impression that diet and drinks exert no influence over the quantity or quality of the urinary secretion, but only that the nitrogenized constituents, especially the Urea, "may fairly serve," to use the language of Dr. Carpenter, "as a measure of the *waste* of the tissues."

The foregoing view of the origin of fibrin also serves to explain the diversity of results which have been obtained by experiments on comparing analytically Arterial with Venous blood, the fibrin being found to predominate sometimes in one and sometimes in the other;—such differences depending undoubtedly on two circumstances, viz: first, the particular vein from which the blood was taken for analysis; and second, on the comparative activity of the *nutritive* and *disintegrative* processes.

If it was the object of this paper to propound any general doctrines, except such as strictly belong to the experimental inquiries above detailed, I should indicate the several steps of assimilation and nutrition by the formation first, of albumen; second, white corpuscles; and third, organized tissue.

While the steps of disintegration or decay, so far as the nitrogenized constituents are concerned, are, first, the formation of fibrin and extractive matter; and second, the various nitrogenized constituents of the excretions.

The generally received doctrine of the formation of all organized structures from cells, and the part which the white corpuscles play in the reparation of injured parts, constituting what M. Travers styles "*the new lymph-bed of organization*," as seen under the microscope, all tend to sustain these views. But it is no part of my present purpose to enter upon a discussion of these interesting topics. And I would by no means present this paper to the profession, until the experiments herein had been further corroborated by repetition and extension, did I not hope that by so doing others would be induced to enter experimentally the same field of inquiry.

